# Musculoskeletal pain among nursing professionals in material and sterilization centers

**DOI:** 10.1590/1980-220X-REEUSP-2023-0019en

**Published:** 2024-01-15

**Authors:** Sérgio Abreu de Jesus, Flaviana Pereira Bastos Nascimento, Gisele Massante Peixoto Tracera, Kayo Henrique Jardel Feitosa Sousa, Katerine Moraes dos Santos, Raphael Sampaio dos Santos, Juliano dos Santos, Regina Célia Gollner Zeitoune

**Affiliations:** 1 Instituto Nacional de Câncer, Rio de Janeiro, RJ, Brazil.; 2 Universidade Federal do Rio de Janeiro, Escola de Enfermagem Anna Nery, Programa de Pós-graduação em Enfermagem, Rio de Janeiro, RJ, Brazil.; 3 Instituto de Atenção à Saúde São Francisco de Assis, Rio de Janeiro, RJ, Brazil.; 4 Universidade Federal do Estado do Rio de Janeiro, Hospital Universitário Gaffrée & Guinle, Rio de Janeiro, RJ, Brazil.

**Keywords:** Working Conditions, Pain, Nursing, Sterilization, Occupational Health, Condiciones de Trabajo, Dolor, Enfermería, Esterilización, Salud Laboral, Condições de Trabalho, Dor, Enfermagem, Esterilização, Saúde Ocupacional

## Abstract

**Objective:**

To identify the presence of musculoskeletal pain during the working day among nursing professionals in material and sterilization centers.

**Method:**

A cross-sectional study with 36 nursing professionals who answered a questionnaire for personal characterization and diagnosis of musculoskeletal disorders and Corlett and Manenica's diagram of painful areas at the beginning and end of the working day. Frequency distribution analysis, Fisher's exact test and likelihood ratio were carried out.

**Results:**

The presence of pain was reported by 80.6% (n = 29) of the participants at the start of the working day and 94.4% (n = 34) at the end, and the prevalence of musculoskeletal disorders was 66.6% (n = 24). There was a statistically significant difference in the number of segments with pain between professionals with and without a diagnosis of musculoskeletal disorders, in the initial and final assessments. The lumbar spine had a higher prevalence of pain in both assessments.

**Conclusion:**

The prevalence of pain increased towards the end of the working day and indicates that there may be a relationship between the work process and the development of pain. It is important to identify working conditions that may contribute to the onset of pain and to adopt preventive measures.

## RESUMO

**Objetivo:**

Identificar a presença de dor osteomuscular durante a jornada de trabalho, em profissionais de enfermagem de centros de material e esterilização.

**Método:**

Estudo transversal, com 36 profissionais de enfermagem, que responderam ao questionário para caracterização pessoal e de diagnóstico de distúrbios osteomusculares e ao diagrama de áreas dolorosas de Corlett e Manenica, no início e fim da jornada de trabalho. Realizou-se análise de distribuição de frequências, teste exato de Fisher e razão de verossimilhança.

**Resultados:**

A presença de dor foi referida por 80,6% (n = 29) dos participantes no início da jornada de trabalho e por 94,4% (n = 34) ao final, e a prevalência de distúrbios osteomusculares foi de 66,6% (n = 24). Houve diferença estatisticamente significativa na quantidade de segmentos com dor entre profissionais com e sem diagnóstico de distúrbios osteomusculares, na avaliação inicial e final. A coluna lombar, em ambas as avaliações, apresentou maior prevalência de dor.

**Conclusão:**

A prevalência de dor aumentou ao final da jornada de trabalho e indica que pode haver relação entre o processo de trabalho e o desenvolvimento de dor. É importante identificar condições de trabalho que podem contribuir para o surgimento da dor e adotar medidas de prevenção.

## RESUMEN

**Objetivo:**

Identificar la presencia de dolor musculoesquelético durante la jornada laboral entre los profesionales de enfermería de los centros de material y esterilización.

**Método:**

Estudio transversal con 36 profesionales de enfermería que respondieron a un cuestionario de caracterización personal y diagnóstico de trastornos musculoesqueléticos y al diagrama de Corlett y Manenica de zonas dolorosas al inicio y al final de la jornada laboral. Se analizaron la distribución de frecuencias, la prueba exacta de Fisher y los cocientes de probabilidad.

**Resultados:**

El 80,6% (n = 29) de los participantes declararon la presencia de dolor al inicio de la jornada laboral y el 94,4% (n = 34) al final, y la prevalencia de trastornos musculoesqueléticos fue del 66,6% (n = 24). Hubo una diferencia estadísticamente significativa en el número de segmentos con dolor entre los profesionales con y sin diagnóstico de trastornos musculoesqueléticos, en las evaluaciones inicial y final. La columna lumbar presentó una mayor prevalencia de dolor en ambas evaluaciones.

**Conclusión:**

La prevalencia de dolor aumentó hacia el final de la jornada laboral e indica que puede existir una relación entre el proceso de trabajo y el desarrollo de dolor. Es importante identificar las condiciones de trabajo que pueden contribuir a la aparición del dolor y adoptar medidas preventivas.

## INTRODUCTION

Musculoskeletal Disorders (MSDs) are a serious public health problem in Brazil and around the world, and identifying situations that may contribute to workers’ MSDs is essential to mitigating health risks^(1–4)^. Data from the European Agency for Safety at Work (OSHA) report show that approximately three out of every five workers in the 28 countries of the European Union (EU) had complaints related to musculoskeletal injuries^(1)^.

In general, healthcare workers suffer more musculoskeletal injuries than other groups of professionals and have a four times greater risk of developing MSDs than those working in the industrial sector^(5)^. In the in-hospital context, nursing stands out for having high levels of musculoskeletal pain with or without comorbidity of MSD^(6,7)^, and the worsening of these conditions can be the result of intense activities, as proven in previous studies^(8)^.

Thus, for this study, Material and Sterilization Centers (MSCs) stood out, whose purpose is to process and supply health products^(9)^ and which, due to the specificities of the work process, such as picking up, washing and transporting heavy boxes of surgical instruments, working for a long time in an orthostatic position, at a fast, repetitive and stressful pace, make professionals more vulnerable to damage to their health^(10)^.

Studies^(6,11–13)^ suggest a relationship between pain, MSD and the work context of the MSC. This is due to the high physical demands of handling heavy materials, the need for agility when processing materials, occupational exposure to contaminated materials, high temperatures and the fast pace of work.

The aim of this study was to identify the presence of musculoskeletal pain during the working day among nursing professionals in a MSC.

## METHOD

### Type of Study

This is a cross-sectional study.

### Study Site

The study was carried out in three class II MSCs, for processing complex and non-complex materials^(10)^, linked to a High Complexity Oncology Center (CACON), located in Rio de Janeiro, RJ, Brazil. These MSCs comprised the following units: hospital unit (HU) 1, made up of 19 nursing professionals in the MSC and serving the specialties of pediatric surgery, thoracic surgery, abdomino-pelvic surgery, urological surgery, head and neck surgery, bone marrow transplantation, neurosurgery and robotic surgery; HU 2, consisting of 16 nursing professionals in the MSC and providing care in the specialties of gynecology and connective bone tissue (orthopedics); HU 3, consisting of 10 nursing professionals in the MSC and providing care in the specialties of mammoplasty and mastology.

### Study Sample

The inclusion criteria were: nursing professionals who had been working in the MSC for at least six months, working during the day and/or night; and the exclusion criteria were: professionals who worked in other sectors, but who eventually worked extra shifts in the unit, professionals who had been temporarily relocated and/or professionals on sick leave during the data collection period.

Based on a census, nine professionals of the 45 eligible nursing professionals were excluded, after applying the aforementioned criteria; six due to prolonged medical leave, two due to administrative leave, as a result of belonging to risk groups for COVID-19, and one due to a sector transfer. In the end, the sample comprised 36 nursing professionals, representing all those who took part in the census.

### Data Collection Instrument

The instrument included a sociodemographic, occupational and health-related conditions questionnaire, as well as Corlett and Manenica’s self-report diagram of painful areas^(14)^.

The questionnaire variables were: a) sociodemographic: age, sex assigned at birth, marital status, ethnicity/color, children and schooling; b) occupational: number of jobs, workload, professional category, time working in the MSC; c) health-related: medical diagnosis of MSD (self-reported).

The questionnaire was assessed for clarity and quality, and a pilot test was carried out with nursing professionals from the MSCs who did not take part in this research, in order to ensure that the information was accurately understood.

Corlett and Manenica’s self-report diagram of painful areas was used to assess the presence, intensity and location of painful complaints^(14)^. It provides an assessment of postural discomfort by means of a map of body regions and allows the identification of areas of discomfort when using the workstation (furniture) studied. The diagram was used in the most up-to-date version, but adapted for this research. In the current version, the “back” region is assessed by the item “back”. In the original version, the back was divided into three areas. For this study, we decided to divide this region into two areas, based on the division in the original version. Thus, the region corresponding to “back” was assessed using the items “dorsal spine” (upper back) and “lumbar spine” (lower back), in order to broaden the options of body areas, making the record more accurate and without causing any damage to applicability. It is an open-access instrument, widely used in ergonomic research in various areas, including nursing.

At the end of the assessment, this instrument provides an overview of the professional’s condition at the time of application, in terms of the presence of painful complaints. Analyzing the intensity of pain was not one of the objectives of this study. To characterize the area of pain, we considered the presence or absence of pain based on the illustrative drawing of the human body proposed in the diagram^(14)^.

To record the location of the pain, the drawing of the human body in a posterior position was used, divided into right and left sides, with 14 symmetrical body segments listed in pairs: 11 for left neck and 12 for right neck, 21 for left shoulder and 22 for right shoulder, 31 for left thoracic region and 32 for right thoracic region, 41 for left lumbar region and 42 for left lumbar region, 51 for left elbow and 52 for right elbow, 61 for left wrist and hand and 62 for right wrist and hand, 71 for right leg and foot and 72 for left leg and foot. The study considered only regions where pain was reported on at least one side (right or left).

### Data Collection

Data collection was carried out by a nurse specialized in operating rooms and sterilization centers, a member of the MSC nursing team at hospital unit 1, from December 2019 to September 2020. The professionals were contacted in person, on a day previously agreed with their immediate supervisor, and collectively received all the information about the study. Those who agreed to take part were given the Informed Consent Form (ICF) and the Data Collection Instrument in a non-transparent envelope. They were first asked to fill in the questionnaire to characterize their profile and then to fill in the diagram of painful areas.

The guidelines for applying this instrument are that it should be done only once, at the end of the workday^(14)^. However, in this study, it was decided to apply it at two times: the beginning and the end of the workday, in order to make more accurate comparisons as to the presence or absence of painful complaints.

In this way, the instruments were filled out as follows: for daytime workers, they were applied at two times, at 7 a.m. and 4 p.m. or 8 a.m. and 5 p.m.; for daytime shift workers, they were applied once, at 7 a.m. and 7 p.m.; for nighttime shift workers, they were also applied once, from 7 p.m. to 7 a.m.; and for 24-hour shift workers, they were applied once, from 7 a.m. on the day the envelope was delivered until 7 a.m. the following day.

As the final approach took place at the end of the working day, it was agreed with the participants and their immediate supervisor that they would have five days from the date of the approach to return the envelope with the completed instruments. When filling in the instruments, the participant marked the number that best represented the intensity of the pain felt in each region marked on the diagram. When there were no painful complaints in a region, a score of zero was recorded. On the day of the instrument collection, all envelopes were readily available.

### Data Processing

The data obtained was entered into Microsoft Excel® spreadsheets, double-entered and validated for comparison and correction in the event of discrepancies. They were then transferred to the Statistical Package for the Social Sciences (SPSS) program, version 21.0, for processing.

Descriptive analyses (frequency distribution) were carried out to identify the profile of the participants and the characteristics related to pain and the diagnosis of MSD. To verify the associations between the presence and location of pain and MSD, as well as the relationships between the number of body segments with painful manifestations and MSD, Pearson’s chi-square test or likelihood ratio or Fisher’s exact test were used. The significance level adopted was 5%.

### Ethical Aspects

This study complied with the ethical precepts of Resolution 466/12 for research involving human beings and used the ICF; it was cleared by the Research Ethics Committees (CEP) of the proposing institutions (Opinion No. 3.636.843/2019) and the co-participating institution (Opinion No. 3.709.457/2019).

## RESULTS

The sample consisted of 36 nursing professionals, three of whom were nurses, 31 technicians and two nursing assistants, predominantly female (83.5%, n = 30), non-white (58.3%, n = 21), educated to high school level (41.7%, n = 15), with children (75%, n = 27), and living without a partner (55.6%, n = 20). The average age of the participants was 47.4 (±9.7) years. Most did not have another job (69.4%, n = 25), worked 40 hours a week (69.4%, n = 25) and had been working in the MSC for between one and five years (55.6%, n = 20). The overall prevalence of self-reported MSD among nursing professionals was 66.7% (n = 24). Painful complaints were observed both at the beginning and end of the working day. Among the participants diagnosed with MSD, 75% (n = 18) reported pain at the initial assessment, while 95.8% (n = 23) reported pain at the final assessment. It should be noted that 91.7% (n = 11) of the participants without a diagnosis of MSD reported pain both at the start and end of the working day (Table 1).

**Table 1:** Painful complaints of nursing professionals at Material and Sterilization Centers and diagnosis of Musculoskeletal Disorders – Rio de Janeiro, RJ, Brazil, 2019-2020. (n = 36)

Musculoskeletal Disorders Diagnosis							
Pain complains	Yes		No			Total	
Start of workday	n	%	n	%	p-value	n	%
Non present	6	25.0	1	8.3	0.384†	7	19.4
Present	18	75.0	11	91.7		29	80.6
**End of workday**							
Non present	1	4.2	1	8.3	1.000†	2	5.6
Present	23	95.8	11	91.7		34	94.4

Source: Research data / †: *Fisher Exact test.*

Table 2 shows the distribution of the location of the painful complaints of nursing professionals with (n = 24) or without (n = 12) a diagnosis of MSD, considering the time of the assessment. Analysis of the results at the two different times of the working day showed an increase in the percentage of those with a diagnosis of MSD in all the body areas analyzed, and in several areas for those without such a diagnosis, although without any statistically significant difference.

**Table 2:** Location of pain, according to the time of evaluation of painful complaints, in nursing professionals from Material and Sterilization Centers, and diagnosis of Musculoskeletal Disorders – Rio de Janeiro, RJ, Brazil, 2019–2020. (n = 36)

	Musculoskeletal Disorders Diagnosis						
Pain location	Yes		No			Total	
Start of workday	n	%	n	%	p-value	n	%
Neck	12	50.0	6	50.0	1.000	18	50.0
Shoulders	12	50.0	5	41.7	0.637	17	47.2
Dorsal spine	12	50.0	6	50.0	1.000	18	50.0
**Lumbar spine**	12	50.0	9	75.0	0.151	21	58.3
Elbow/forearm	6	25.0	3	25.0	1.000†	9	25.0
Wrist/hand	11	45.8	3	25.0	0.292†	14	38.9
Leg/Foot	14	58.3	6	50.0	0.635	20	55.6
**End of workday**							
Neck	17	70.8	6	50.0	0.281†	23	63.9
Shoulders	16	66.7	7	58.3	0.720†	23	63.9
Dorsal spine	15	62.5	6	50.0	0.473	21	58.3
Lumbar spine	20	83.3	9	75.0	0.664†	29	80.6
Elbow/forearm	7	29.2	4	33.3	1.000†	11	30.6
Wrist/hand	12	50.0	5	41.7	0.637	17	47.2
Leg/Foot	20	83.3	9	75.0	0.664†	29	80.6

†: *Fisher Exact test.*

Among the professionals diagnosed with MSD, there was an increase in the number of segments with pain, as shown in Table 3. It is noteworthy that in the final assessment, compared to the initial assessment, 8.7% (n = 2) of the professionals had pain in two body segments and 21.7% (n = 5) had pain in four body segments; and 21.7% (n = 5) had pain in six body segments. The p-value was significant, showing a strong relationship between pain in the body segments and musculoskeletal disorders. It should be noted that it was not necessarily the case that all the participants had pain at both times or in all the segments investigated.

**Table 3:** Number of segments with pain, according to the time of evaluation of painful complaints, in nursing professionals from Material and Sterilization Centers, and diagnosis of Musculoskeletal Disorders – Rio de Janeiro, RJ, Brazil, 2019–2020. (n = 36)

	Musculoskeletal Disorders Diagnosis						
Number of segments with pain	Yes		No			Total	
Start of workday (n = 29)	n	%	n	%	Valor de p‡	n	%
1	3	16.7	1	9.1	**0.018**	4	13.8
2	0	0.0	4	36.4		4	13.8
3	3	16.7	3	27.3		6	20.7
4	2	11.1	0	0.0		2	6.9
5	5	27.8	0	0.0		5	17.2
6	1	5.6	1	9.1		2	6.9
7	4	22.2	2	18.2		6	20.7
**End of workday(n = 34)**							
1	1	4.3	0	0.0	**0.011**	1	2.9
2	2	8.7	5	45.5		7	20.6
3	2	8.7	0	0.0		2	5.9
4	5	21.7	2	18.2		7	20.6
5	5	21.7	0	0.0		5	14.7
6	5	21.7	0	0.0		5	14.7
7	3	13.0	4	36.4		7	20.6

‡: Likelihood ratio.

## DISCUSSION

The research evidenced that the presence of painful symptoms and a diagnosis of MSD among nursing professionals is a concern in the Material and Sterilization Centers investigated, corroborating previous studies that have also demonstrated this issue in other professional practice settings^(6–8,15–17)^.

Studies show that nursing professionals are at high risk of MSD, owing to their work dynamics and, as a result of this wear and tear, they end up suffering from painful sensations during or at the end of the working day. This is exacerbated when they perform activities involving repetitive movements, forced postures, standing and/or walking, as well as working in an unfavorable work environment, with sudden movements, weight and emotional stress. The findings of this study also corroborate the fact that professional health practice favors exposure of the musculoskeletal system and, consequently, the appearance of pain in various segments of the body^(12,18, 19, 20)^.

Research^(13,16,17)^ indicates that musculoskeletal disorders and pain are the main causes of absenteeism and sick leave among nursing professionals. A study^(17)^ investigating 2.761 absences among nursing professionals at a university hospital in Rio Grande do Sul, Brazil, found that MSDs were the main cause, accounting for 16.26% (n = 449) of absences. This total included 220 (9.94%) professionals, of whom 48.9% had more than one absence, demonstrating the persistence of illness.

The data presented so far is in line with that obtained in this study and in another national study^(17)^. From this point of view, it reinforces the understanding that areas of greater repetitiveness and support of body weight have greater evidence for the appearance of MSD and painful sensations in nursing professionals.

These inferences can be transposed to the context of working in the MSC. Nursing professionals in this sector often report painful sensations, probably associated with the repetitiveness of activities involving washing materials, carrying heavy boxes and baskets with materials prepared for sterilization, as well as transporting sterile materials to be dispensed to consumer units. As a result, they are a group of workers prone to developing occupational stress and illness related to the occupational risks inherent in the sector^(21)^.

A study^(22)^ carried out in Ecuador with MSC nursing professionals confirms the data collected here, as it showed that 75% of the professionals reported making sudden movements and 42% reported feeling stressed during the working day, which contributed to the prevalence of painful areas in the shoulders and back, followed by pain in the neck, arms and waist.

It was found that 75% of the MSC nursing professionals investigated reported pain at the beginning of their shift, and 95.8% said they felt pain at the end of their shift. This suggests that after physical, organizational and cognitive exposure to the work in the sector, the professionals responded to the stimuli through painful sensations. It is therefore possible to associate these pains with MSD, which in the long term can lead to work incapacity^(1,23)^.

The regions most affected by painful sensations were the lumbar and dorsal spine, feet/legs, neck and shoulders, both at the beginning and end of the working day. These data are corroborated by studies^(24,25)^ which identified a higher prevalence of pain in the lumbar region, followed by the neck and shoulder in nurses, nursing technicians and nursing assistants in the material and sterilization center and emergency department.

Corroborating these findings, the prevalence of MSD in a sample of 1.932 Chinese nurses was 79.52%, with painful symptoms in the waist region (64.83%), neck (61.83) and shoulders (52.36%)^(13)^. In Iran, among 211 nurses aged 35–45, the most affected regions were the lumbar (88.33%), knee (83.33%), femoral (71%) and neck (55%)^(16)^.

Analysis of the data showed that nursing professionals started their working day with pain, even those who were not diagnosed with MSD. This is worrying from the perspective of the health of these workers. In addition, the routine of working in the MSC first thing in the morning contributes to overload and may be related to the early onset of pain. Another factor that cannot be overlooked is the postures adopted by the workers. What’s more, in practice, the volume of demands in the morning is more intense. This is when the greatest number of procedures and surgeries take place, requiring the sector to be more agile and prompt in preparing and releasing material throughout the day.

In the final assessment - at the end of the shift - the results for the neck, shoulder, lumbar spine and wrist/hand regions remained similar to those of the first assessment, which shows the continued use of these structures and the consequent painful sensation from repetitive movements. However, there was an increase in the dorsal column, elbow/forearm and leg/foot.

These results contribute to the understanding that, at the end of the working day, professionals end up feeling pain. It can be inferred that this pain is triggered by fatigue due to the constant use of the back and elbow/forearm throughout the working day, during activities which involve cleaning, drying, transporting heavy materials, as well as preparing trays in a sitting or standing position, using the upper limbs to fold, check and transport them to the sterilization sector.

This is concurring with other studies^(26–28)^ regarding the health of MSC workers, which state that ergonomic risks are present in this work environment and can affect productivity and quality of care. In addition, there is a high prevalence of MSD and migraines.

In the case of the lower limbs, studies corroborate that the legs and feet are responsible for supporting the body and, consequently, long periods of standing can lead to painful sensations. Reports of exhaustion due to maintaining the same posture for long periods and physical exertion are recurrent in this group^(27,28)^. The results of the study in question corroborate these observations.

Exemplifying this situation, a study^(29)^ found a high prevalence of MSD associated with standing for long periods and manual handling of materials during the working day among nurses in Greece. Another study^(30)^ reinforces this finding and adds that physically demanding work, involving the adoption of incorrect postures, contributes greatly to the development of MSD and the presence of pain.

Thus, reports of pain in various regions of the body are common among nursing professionals, indicating that this class has a clear prevalence of pain and, consequently, work limitations associated with the work environment, repetitive movements, inappropriate postures, stress and a fast pace of work^(1,7,30)^. These situations, combined over the long term, can compromise the worker’s functional capacity and lead to permanent musculoskeletal injuries.

It should therefore be pointed out that working with pain limits the professional’s work, making it stressful and demotivating. Just one area affected by pain can have serious consequences for a professional’s health. It is therefore difficult for the professional to continue working with several areas of pain at the same time, as identified in this study.

The limitations of this study are related to its typology, which makes it impossible to establish causal relationships, and to the fact that the professionals may have answered according to what was expected, rather than according to their intentions, which may have underestimated or overestimated the outcomes, which was enhanced by the self-administered form of the instrument.

On the other hand, the study advances scientific knowledge by investigating a field of nursing that is still invisible, but which has an impact on the whole process of caring for individuals. For a long time, MSCs were located in isolated areas of Healthcare Establishments, with no contact with other sectors, as they are a closed environment in which the careful adoption of safety measures is essential. However, this has led to a distancing in the imagination of the institutions, which has generated a lack of knowledge of the role and importance of the activities carried out there for other members of the staff. This has also been reflected in the absence or incipient concern of researchers in developing research involving issues specific to that environment. Thus, this study fills a gap in the production of scientific knowledge, especially in relation to the processes of illness of this public, broadening the contributions by pointing out not only the presence of MSD and pain, but also the moments when these episodes occur.

This article highlights the need to identify early on the potential risks of illness among nursing professionals who work in MSCs, as well as those professionals who are most vulnerable, in order to devise strategies to mitigate the damage that may eventually occur to their health. This shows the importance of shared action between managers and workers to promote better working conditions, especially when it comes to painful complaints and MSD, management and prevention programs for ergonomic risks, which are intrinsically related to this type of illness.

As this was an exploratory study, it did not delve into the analysis of MSDs at this initial stage. The population was specific, which prevents generalizations, and no differential diagnosis was made for acute and chronic pain. These limitations should be overcome in new studies.

The study highlights the importance of identifying the working conditions that may be contributing to the onset of pain and the need to adopt preventive measures. This could include training in ergonomic techniques, the use of support equipment or changes to the design of the workplace. It contributes to raising awareness among managers, the creation of public policies related to the prevention of MSD and pain in MSC nursing professionals, as well as treatments for those already affected; it favors concrete data that can guide practical interventions, such as rotations between professionals in the WEC areas, regular breaks and stretching exercises, for example.

## CONCLUSION

The presence of pain was reported by 29 (80.6%) of the participants at the beginning of the workday and by 34 (94.4%) at the end. The overall prevalence of MSD among nursing professionals was 66.6% (n = 24). Among the professionals diagnosed with MSD, there was an increase in the number of participants reporting pain at the end of the shift. These data indicate that there may be a relationship between the work process and the development of pain, identified and verbalized by the participating professionals.

The region with the highest prevalence of pain at both assessment times was the lumbar spine, followed by legs/feet, dorsal spine and neck in the initial assessment. In the final assessment, the regions with the highest prevalence of pain were the legs/feet, neck and shoulders. At the end of the work shift, compared to the first assessment, there was a quantitative increase in the number of body segments in pain.

Finally, the increase in the prevalence of pain among nursing professionals after the working day highlights the importance of paying attention to all areas of the body and the urgent need for interventions in workers’ health. The continuous presence of pain related to musculoskeletal overload can increase the risk of developing MSD related to the dynamics of the service.
