# ERRATUM: Musculoskeletal pain among nursing professionals in material and sterilization centers

**DOI:** 10.1590/1980-220X-REEUSP-2023-0019eren

**Published:** 2024-04-19

In the article “Musculoskeletal pain among nursing professionals in material and sterilizantion centers”, with the DOI: https://doi.org/10.1590/1980-220X-REEUSP-2023-0019en, published at: Revista da Escola de Enfermagem da USP [online], v. 57: e-20230019, on page 8 include the section:

Financial support

This study was financed in part by the Coordenação de Aperfeiçoamento de Pessoal de Nível Superior – Brasil (CAPES) – Finance Code 001.

